# 

**Published:** 1914-03

**Authors:** 


					﻿indexed g
OFFICIAL ORGAN—State Society Social Hygiene and Eugenics.
W.XMX
MARCH, 1914
No. 9
Red BcJ>”
TEXAS MEDICAL JOlj^UI
ESTABLISHED JULY, 1885
PUBLISHED MONTHLY AT AUSTIN, TEXAS, BY
L E. DANIEL,. M. D., - - -
, PRINTED THE VON BOECKM ANN-JONES CO.
Subscription, Si. 00 aYear, in Advance.	-	-	- |SiyggC^es^H|^e^U \
Entered at the Postoffice at. Austin, Texas, as second class riaail mattei
fl
e
8
■
Z'
fl
'W
1
				

